# Predicting the invasiveness of alpine newts in the UK

**DOI:** 10.1007/s10530-025-03543-2

**Published:** 2025-03-10

**Authors:** Alexandra C. North, Luke J. Sutton, Jason L. Brown, Trenton W. J. Garner, Richard A. Billington, John W. Wilkinson, Manuela Truebano, Robert Puschendorf

**Affiliations:** 1 University of Plymouth, Plymouth, UK; 2 Southern Illinois University, Carbondale, IL USA; 3 Institute of Zoology, Zoological Society London, London, UK; 4 Amphibian and Reptile Conservation Trust, Bournemouth, UK

**Keywords:** Niche, Invasive, Species distribution modelling, Maxent, Ichthyosaura alpestris, Alpine newt

## Abstract

Predicting invasion risk to novel environments is essential for risk management and conservation decision making but the evolutionary lineage at which to make these predictions is often unclear. Here we predict the current suitability across the United Kingdom (UK) for the alpine newt *Ichthyosaura alpestris*, a species with a complex evolutionary history, a broad native range, a growing number of introduced populations and anecdotal reports of ecological consequences to native amphibian communities. We use species distribution and ecological niche modelling to predict environmental suitability of the alpine newt in the UK at both the species-level and lineage-level and to quantify evolutionary lineage niche overlap. We show good model transferability at the species-level and parts of the UK—especially central and eastern England and parts of central and northern Scotland—to be highly environmentally suitable for the alpine newt. Our findings provide evidence of environmental niche differences at the lineage-level, with the Greek lineage being distinct from most other lineages, but with low confidence in maxent predictions for the Greek, Balkan and Italian lineages due to high levels of extrapolation. In contrast, the niche of the UK records appear to share the same niche as the Central lineage. We find 66% of currently known alpine newt records to fall within areas predicted to be environmentally suitable at the species-level, providing a series of testable hypotheses to better understand the invasion ecology of this species in the UK.

**Supplementary Information:**

The online version contains supplementary material available at 10.1007/s10530-025-03543-2.

## Introduction

An understanding of the risks of introduction, establishment, spread and impact are critical to building effective risk assessments for invasive species (Roy et al. 2018). Predicting current and potential distributions in particular can be used to prioritise decision making; streamlining detection and management is especially useful since invasive species management is already estimated to cost US$1.4 billion per year worldwide (1970–2017; Diagne et al. 2021). Understanding an organism’s abiotic niche is especially relevant to predicting establishment risk for invasive species (Keller et al. 2009). Such abiotic requirements are commonly determined using correlative models, which estimate statistical relationships between species occurrence records and environmental data. This approach uses ecological niche or species distribution models (SDMs) and is frequently applied in invasion ecology (Srivastava et al. 2019).

Biogeographic features, evolutionary processes, and environmental factors play a key role in niche evolution and divergence (Kozak et al. 2008; Calatayud et al. 2019). Whilst closely related phylogenetic groups may respond similarly to environmental gradients (i.e. niche conservatism) (Pyron et al. 2015), in other cases, spatial environmental heterogeneity alongside geographic isolation or limited dispersal ability can accelerate local adaptation (Smith et al. 2019). Despite evolutionary processes often operating at smaller geographic scales than that of the whole range of a species, until more recently evolutionary relationships had been given little consideration when estimating the niche (Smith et al. 2019).

Ignoring evolutionary relationships can lose important information; species distribution predictions of the wide ranging oldfield mouse *Peromyscus polionotus,* for example, were improved by splitting data into subspecies compared to geographically-based units or pooled data across their distributional range, as the subspecies approach accounted for biological processes at play (Gonzalez et al. 2011). Similarly, improved invasion predictions were made when utilising biologically relevant units of distribution records compared to the species as a whole for the introduced Siberian chipmunk *Eutamias sibiricus* worldwide (Mori et al. 2019), invading *Dendroctonus* bark beetles in China (Godefroid et al. 2016), and ring-necked parakeets *Psittacula krameri* in Europe (Strubbe et al. 2015b; Cardador et al. 2016). This could have profound implications for risk assessments since invasion potential could be predicted differently depending on geographic origin of the invader.

Amphibians are amongst the most threatened taxonomic group worldwide (González-del-Pliego et al. 2019; IUCN 2020), yet they can also be highly successful invaders (Kraus 2015). One such example is the alpine newt *Ichthyosaura alpestris*, a small-bodied urodele that is declining in parts of its range (Denoël et al. 2005a, 2019) but is considered an introduced species elsewhere (Bell and Bell 1995; Blackwell 2002; Bond and Haycock 2008; Arntzen et al. 2016; Bell 2016; Palomar et al. 2017; Jakóbik et al. 2019). Its native range of mainland Europe is geographically and altitudinally broad (Arntzen et al. 2009), covering three prominent glacial refugia (Schmitt 2007). The most comprehensive multi-marker phylogenetic analyses of the species suggest that the alpine newt consists of four evolutionary lineages, covering central Europe, Italy, Greece and the rest of the Balkans (Recuero et al. 2014).

Complex evolutionary history alongside geographically varied life histories and morphology (Denoël et al. 2001; Ivanović et al. 2009; Vukov et al. 2011) could be indicative of niche differences below the species level for the alpine newt. This could be of relevance for conservation management and policy since this species has successfully invaded New Zealand (Arntzen et al. 2016; Bell 2016), various locations across mainland Europe (Arntzen et al. 2016; Palomar et al. 2017; Jakóbik et al. 2019) and the United Kingdom (UK) (Bell and Bell 1995; Blackwell 2002; Bond and Haycock 2008). In the UK, over 100 geographically independent sightings have been reported since the species was first detected in the 1920s (Bell and Bell 1995; Blackwell 2002; Bond and Haycock 2008). Whilst their ecological impact has yet to be formally quantified, research is ongoing and anecdotal reports are indicative of context-dependent ecological consequences for native species through potential predation, competition or disease transfer (Winchester 2016; Bell 2016; Graham and Togridou 2017, 2018).

An understanding of how suitable the UK is likely to be for the alpine newt is therefore an important exercise for conservation decision-making. Here, we aim to (1) ascertain the environmental suitability of the UK for the alpine newt, (2) determine whether invasive potential differs by evolutionary lineage and (3) ascertain whether any differences in lineage invasive potential are due to environmental niche divergence. We first predict environmental suitability at both the lineage and species-level using species distribution models, with a focus on understanding whether invasion risk differs by lineage. We ascertain the number of known alpine newt records that fall within areas predicted to be of high environmental suitability based on these models. We then quantify niche overlap between evolutionary lineages using niche overlap and niche divergence tests to determine if any differences in predicted environmental suitability are due to niche divergence or other factors. We provide insights into the feasibility of environmental-based risk analysis for a species within a taxonomic group that is typically underrepresented in the SDM literature (Feldman et al. 2021).

## Methods

### Distribution records

#### Native range occurrence data

The phylogeography of *Ichthyosaura alpestris* suggests that each presence record can be broadly assigned to a lineage based on its geographic location (Recuero et al. 2014). These correspond to (1) the subspecies *I. a veluchiensis*, which we refer to here as the Greek lineage (2) subspecies *I. a. apuana* and *I. a. inexpectata* which we refer here to as the Italian lineage (3) subspecies *I. a. alpestris* central European populations and *I. a. cyreni* which we refer here to as the Central lineage and (4) subspecies *I. a. alpestris* eastern European populations and *I. a. montenegrina* which we refer to here as the Balkan lineage. Research-grade distribution records were obtained from a number of sources including the Global Biodiversity Information Facility (human observation data only) (GBIF 2024), iNaturalist (downloaded March 2024), contacts of authors and local record centres and a literature review. The literature review was conducted in June 2020 and updated in April 2024 using Web of Science and the keywords *alpine newt* OR *Ichthyosaura alpestris* OR *Triton alpestris* OR *Triturus alpestris* OR *Mesotriton alpestris* (n = 494 citations, n = 98 contained spatial information, n = 90 were inaccessible). The global standard WGS84 (EPSG 4326) was assumed for coordinates obtained from the literature when datum was not specified, but these were spot-checked against locality descriptors from the source.

#### Invaded range occurrence data

Invaded range data for the UK was obtained through a series of data repositories (Record Pool, NBN Atlas, ARC; accessed originally in May 2020 and updated in March 2024), the same systematic literature search as above, expert contacts and a social media campaign launched in 2020 that solicited up to date records from across the UK. New records received from members of the public were validated based on the expert-level of the participant submitting the record (i.e. a known newt expert or professional ecologist) or the presence of photographic evidence.

### Occurrence data cleaning

**Fig. 1:**
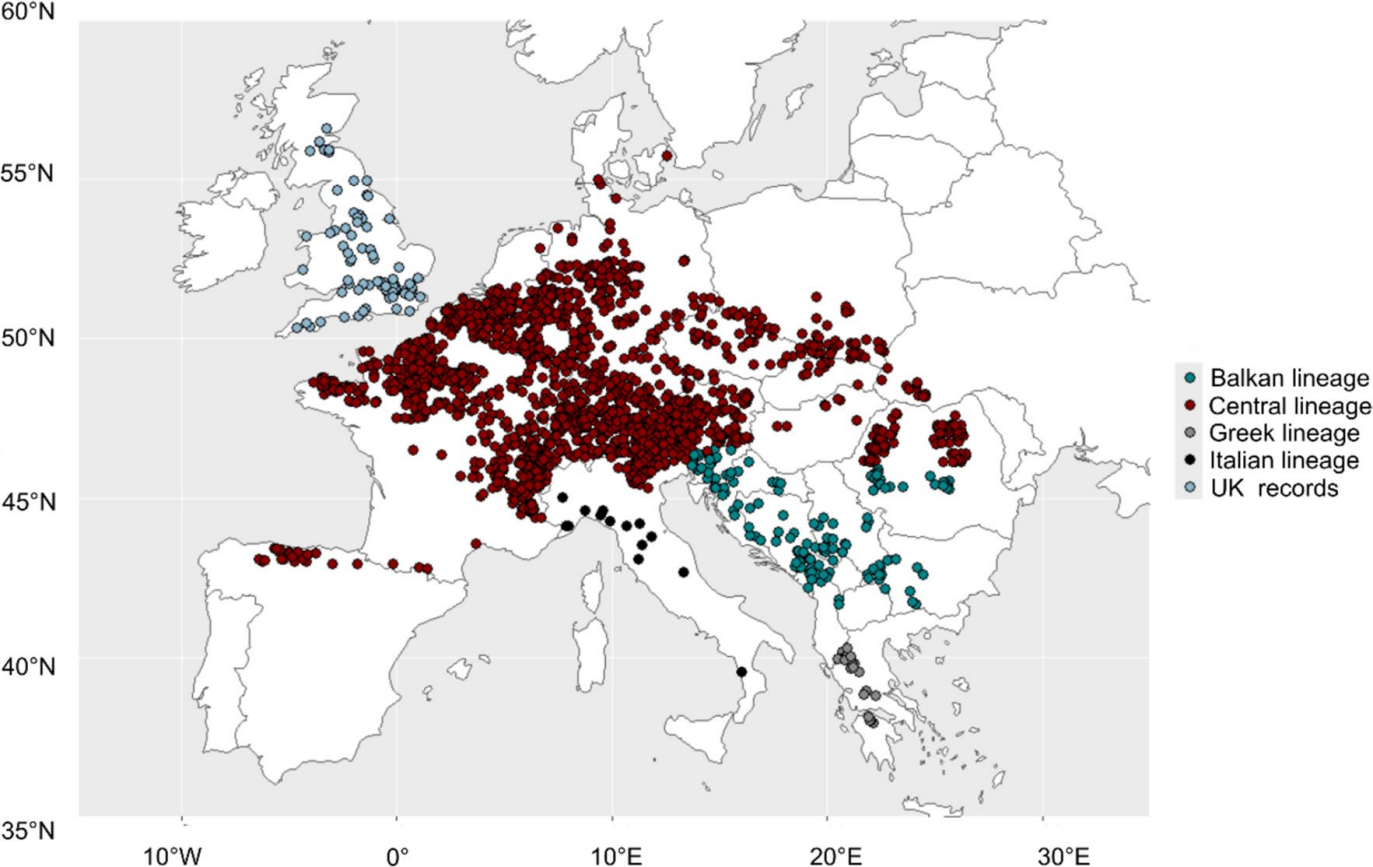
Rarefied *Ichthyosaura alpestris* occurrence records utilised in this study, coloured by evolutionary lineage as geographically defined by Recuero et al. (2014), alongside UK records

GBIF data that lacked information on coordinate certainty were removed, and the remaining data were crosschecked so that all remaining records had at least two decimal places and specified a positional accuracy of 1000m or greater. For data within the literature, certainty was assigned according to the specified number of decimal places only. Repository data with known spatial issues or flags and unknown or questionable origins were excluded to ensure that captive and obviously incorrect records (i.e. occurrences over the sea, museum preserved specimens, fossil records) were omitted from the final dataset. All data were transformed into WGS84 (EPSG 4326) and records before 1970 (or of unknown date) were excluded to ensure occurrence data matched the timescale and projection of the environmental datasets. The resulting dataset contained 10,012 records and these were spatially rarefied using a 5 km spatial filter for all subsequent analyses to match the resolution of the environmental data. Spatial thinning was conducted independently for each dataset, resulting in 1842 occurrence points for the central lineage, 143 records for the Balkans, 21 records for Greece, 14 for the Italian lineage, 75 for the UK records and 2027 for the species-level (all native lineages combined) analysis (Fig. 1 for final dataset).

### Environmental data

A suite of climatic, hydrological, topological and land cover variables of biological relevance to amphibians were initially considered for analysis. Climatic, hydrological and topographic data were obtained from WorldClim (v2.1; Fick and Hijmans 2017) (https://www.worldclim.org/) and ENVIREM (Title and Bemmels 2018) (https://envirem.github.io/) global datasets. Worldclim data spanned the time period 1970–2000, ENVIREM data spanned 1960–1990 and both were obtained at a 2.5 min resolution (equating to ~ 5 km cells). Consensus land cover variables were obtained from EarthEnv (Tuanmu and Jetz 2014) (https://www.earthenv.org/landcover) at 1km resolution and were resampled to match the resolution of other datasets. Variables included annual mean temperature (WC1), temperature seasonality (WC4), maximum temperature of the warmest month (WC5), minimum temperature of the coldest month (WC6), annual precipitation (WC12), precipitation seasonality (WC15), precipitation of the wettest (WC16), and driest quarter (WC17), climatic moisture index (CMI), topographic wetness index (TWI), deciduous broadleaf cover, mixed tree cover and herbaceous plant cover. These were considered important in defining the width of ambient temperatures experienced in the environment (relevant to ectothermic newts), energy inputs into the system (influencing vegetation growth and subsequent terrestrial habitat availability important to alpine newt life cycles), hydrological processes and overall moisture availability and variation (critical to a water-breeding species) and variability in temperature (influencing thermoregulation and subsequent habitat selection) (Denoël 2004; Kopecký et al. 2010; Balogová and Gvoždík 2015). Deciduous broadleaf, mixed tree and herbaceous plant cover were considered important terrestrial habitat providing invertebrate-rich foraging and varied sheltering opportunities (e.g. deadwood, leaf litter and plant understory) (Denoël and Ficetola 2008; Vuorio et al. 2015).

**Fig. 2:**
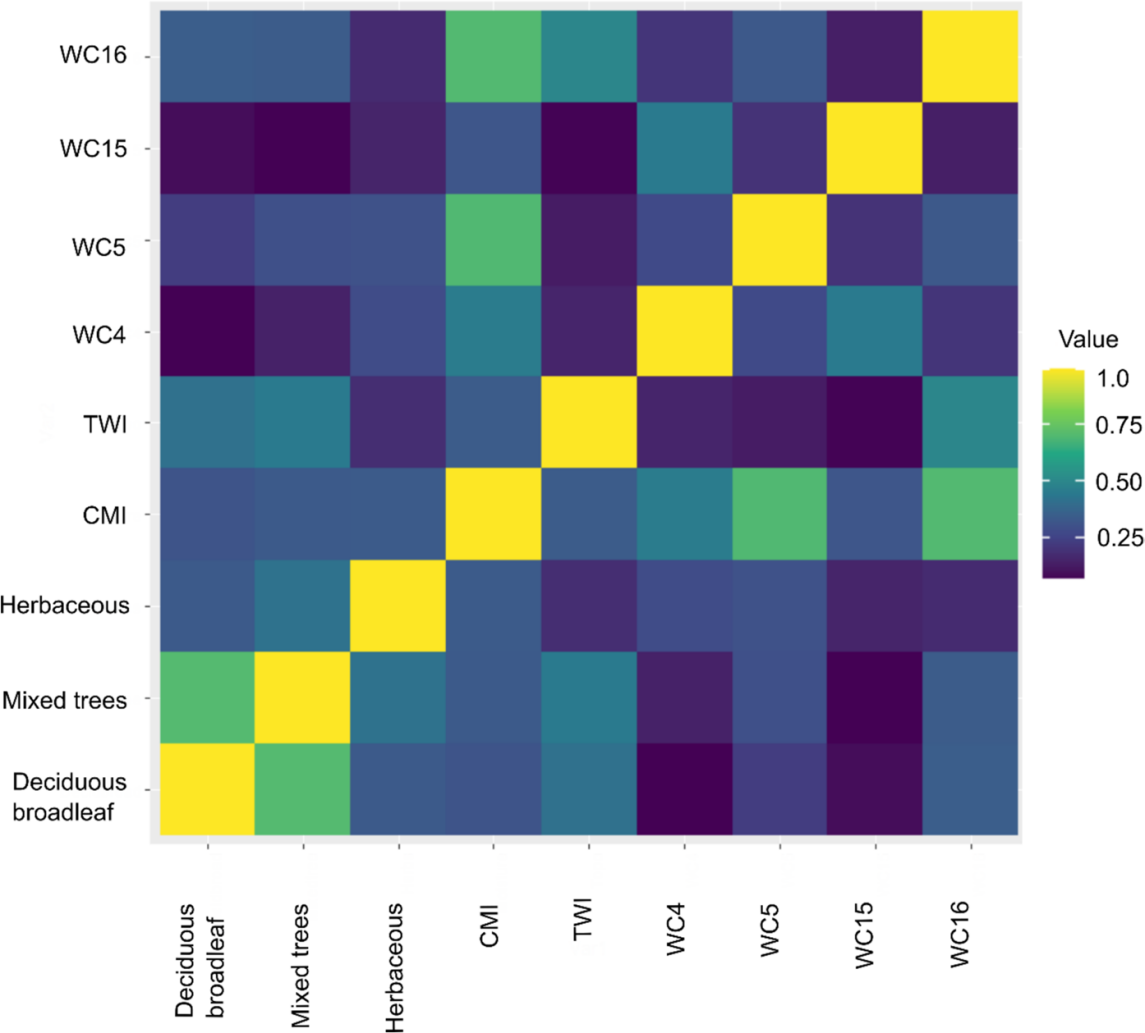
Spearman rank correlation coefficient matrix for the 9 environmental variables included in the final analyses. Values closer to zero depict low correlation, and yellow depicts complete correlation

An expert-driven approach to variable selection has been recommended above data-driven as standard (Santini et al. 2021) and common practise is to remove highly correlated variables. Multi-collinearity of predictor variables were therefore checked using Variance Inflation Factor (VIF) analysis (using package usdm; Naimi et al 2014) and Spearman correlation coefficients. A threshold of 10 (VIF) and 0.74 (spearman correlation) was utilised for variable inclusion and this resulted in a final dataset of nine variables with low collinearity (see Fig. 2): WC4, WC5, WC15, WC16, deciduous broadleaf cover, mixed tree cover, herbaceous plant cover, CMI and TWI. The same temporal and spatial resolution data were used for both building and transferring the model.

### Species distribution modelling

Environmental suitability of the UK for each *I. alpestris* evolutionary lineage was modelled and compared to a prediction at the species-level using a new formulation of Maxent species distribution models—maxnet (Phillips et al. 2017). Data were partitioned into geographic folds using ENMeval (Kass et al. 2021) and all model training and evaluation was conducted using the R package SDMtune (Vignali et al. 2021). Geographic partitioning increases independence between training and testing datasets with block partitioning being recommended for applications predicting across time or space (Muscarella et al. 2014). Combinations of 0.1–2.9 regularisation were tested with 0.2 intervals (Vignali et al. 2021) and the best ranked model (according to AUC) was used to train and predict our final models into the UK. Feature classes (or response functions) were tested based on sample size of occurrence records, with linear, quadratic and hinge feature classes being tested for the Greek and Italian lineages, as these are appropriate for sample sizes of 15–79 (Phillips and Dudík, 2008). For Balkan, Central and species-level analysis, product features were also tested as these are suitable when occurrence records > 80 (Phillips and Dudík 2008). Feature classes constrain predicted distributions according to the environmental data at each occurrence locality and are used to build response curves that can be non-linear (Phillips et al. 2017).

Maxnet uses the principle of maximum entropy on presence only datasets (Phillips et al. 2006) but has been recently shown to be mathematically equivalent to an inhomogeneous Poisson process which is the most appropriate modelling framework for SDMs using presence-only data with small sample sizes (Hernandez et al. 2006; Phillips et al. 2017). Occurrence records were spatially rarefied to 5 km as such systematic sampling has been shown to be most effective in reducing sampling bias across a range of scenarios (Fourcade et al. 2014). Study areas were selected using a 200km buffered minimum convex polygon around occurrence records and due to varying sizes of study extent across lineages (and therefore differing numbers of available pixels for selection), the number of background points selected per lineage was calculated at 20% of the available environmental pixels to avoid model overfitting (Barbet-Massin et al. 2012). This resulted in 1779 background points for the Greek lineage, 10,631 for the Balkan, 3866 for the Italian lineage, 36,495 for the Central lineage and 42,406 for the species-level.

Two evaluation metrics were utilised, the threshold-independent Area Under the Curve (AUC) (with good model discrimination being defined between 0.7 and 0.9; Pearce and Ferrier 2000) and threshold-dependent TSS (True Skill Statistic) (good model discrimination being defined at > 0; Allouche et al. 2006). These metrics were reported for the top optimised model (^tm^) as well as block-cross validated training (^trainingBC^) and testing datasets (^testingBC^). An additional metric ^AUCdiff^ was calculated as the difference between the AUC for training and testing datasets, as a measure of model over or underfitting (Low et al. 2021). The final maps presented are based on complementary log–log (Cloglog) predictions based on the highest-ranking model with clamping limiting extrapolation outside of environments experienced during data training to produce more realistic predictions (Stohlgren et al. 2011). Cloglog is an index of occurrence probability (or environmental suitability) ranging from 0 to 1 (Phillips et al. 2017), with values of one indicating highest suitability and zero lowest suitability. Multivariate Environmental Similarity Surface (MESS) maps were created to determine where models were extrapolating and where, therefore, less confidence should be put onto final predictions (Elith et al. 2010; Srivastava et al. 2021). Negative values indicate extrapolation beyond the range in predictors used in model calibration. The sensitivity–specificity sum maximization thresholding approach was applied to the final models to predict presence-absence and to calculate the percentage of known UK alpine newt records (rarefied to 1 km to increase independence of populations) that fell within areas predicted to be suitable. This thresholding approach has been shown to have low rates of false positive and negatives (Liu et al. 2005) and is most appropriate for presence-only data analyses (Liu et al. 2013, 2016).

### Niche analysis

Pairwise analyses were conducted to determine the level of occupied niche similarity between each of the native evolutionary lineages as well as with the most up to date distribution (and therefore predicted niche) of the non-native UK populations. Analyses focussed solely on comparing environmental opposed to geographic space because environments are unlikely to be equally available to each lineage geographically, and geographic analyses are often biased towards the most prevalent land cover types (Brown and Carnaval 2019). Niche Overlap Tests (NOT) and Niche Divergence Tests (NDT) were implemented utilising equivalence and background tests. The equivalence test utilises the Schoener’s D statistic to determine niche similarity with values ranging from zero to one. Zero depicts complete niche divergence and one depicts niche equivalency. Background tests act as a type of power analysis and determines how capable the equivalence test is in detecting differences considering the environmental space that is available (Brown and Carnaval 2019). The background test was conducted twice per pairwise analysis to compare the similarity between lineage 1 and a random shifting of lineage 2 in geographic space, and vice versa (Brown and Carnaval 2019). Two significant background tests reveal high discriminatory power in the analysis, one significant background test signifies some power and no significant background tests indicate no statistical power for the equivalence findings. Equilibrium of current distributions was not assumed as this is considered to be an unrealistic state for most species’ distributions (Brown and Carnaval 2019), including amphibians across Europe (Araújo and Pearson 2005). The NOT was calculated utilising all available environments whereas the NDT utilises only analogous environmental space (i.e. only environmental space that is shared by both lineages). The use of both NOT and NDT helps to determine whether any niche differences identified are a result of access to different environmental conditions, differences in life histories and/or ecological interactions (NOT), or true divergent evolution (NDT). Niche analyses were corrected according to the abundance of the environmental conditions available within the range of each lineage, as this has been shown to improve model estimates (Brown and Carnaval 2019).

Principal Component Analysis was utilised to reduce the variables to the first two principal components and trimmed according to accessible environmental space. Conservative kernel smoothing values of two were utilised and thresh.espace.z values were set at 0.0001. Accessible environmental space for each of the lineages was calculated using a 200km buffered radius around each of the lineages occurrence records. The default resolution of 100 was used to create a gridded continuous environmental surface for analysis, with 100 balancing good resolution against computational power. The number of iterations for equivalence and background statistics was set at 250 (Brown and Carnaval 2019). Niche overlap analyses were conducted in R studio version 2022.7.2.576 using R version 4.2.1 (R Core Team 2022) and the package Humboldt (Brown and Carnaval 2019) that builds upon methods initially developed by Broennimann et al. (2012), Petitpierre et al. (2012) and Qiao et al. (2017).

## Results

### Predicted environmental suitability of I. alpestris lineages in the UK

**Fig. 3:**
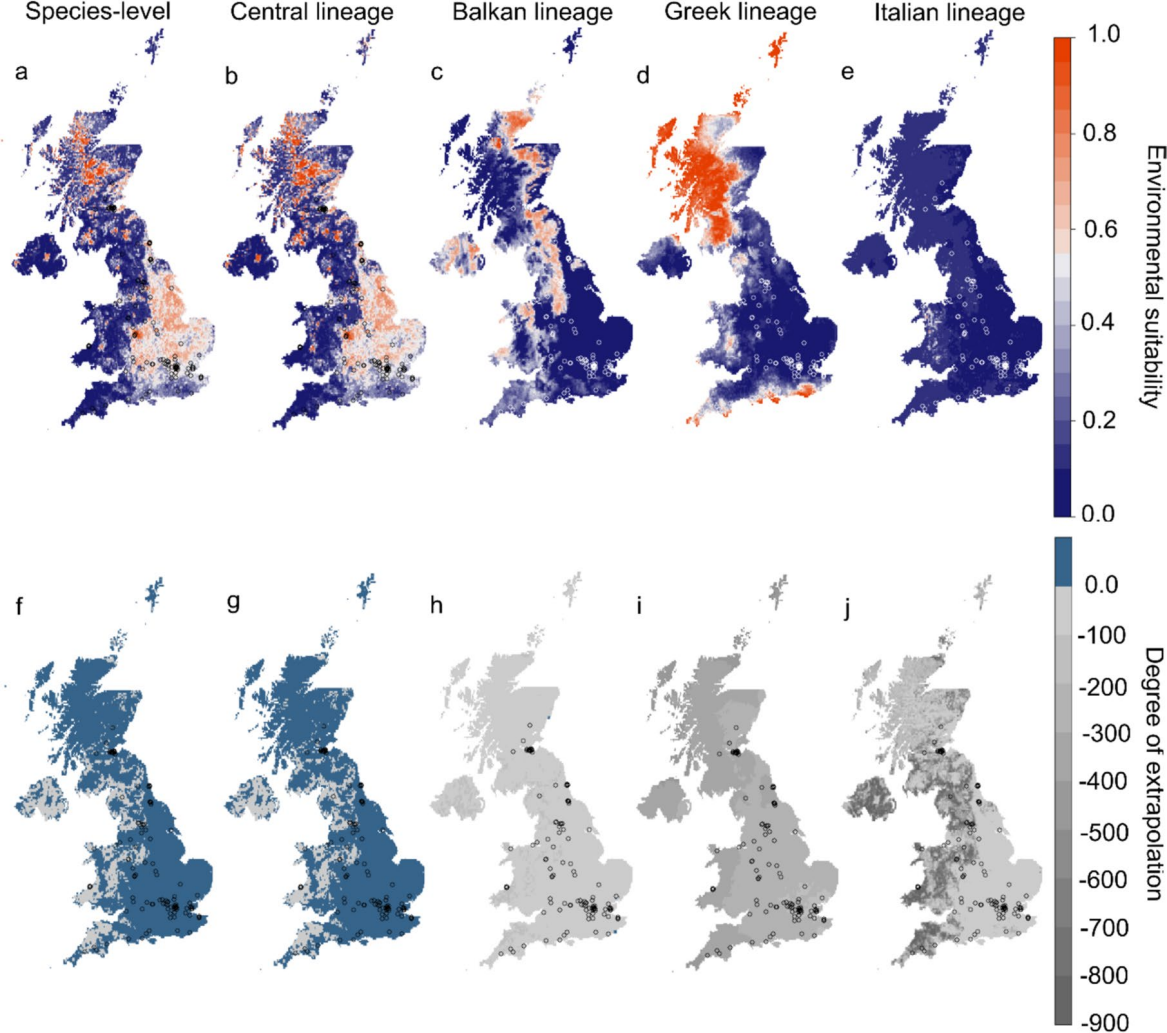
**a**–**e** Cloglog index of occurrence probability shows predicted environmental suitability across the UK based on the environmental niche for all modelled lineages combined (species-level) as well as each lineage separately. Warmer colours depict higher environmental suitability and colder colours depict lower environmental suitability f–j MESS maps show regions vulnerable to extrapolation and areas therefore that we can be less confident in predictions. Shades of grey represent areas of extrapolation, with darker greys representing higher degrees of extrapolation. Areas of blue represent values above zero which represent no extrapolation. Known UK populations (rarefied to 1km to ensure independence) are overlayed as open circles

**Fig. 4:**
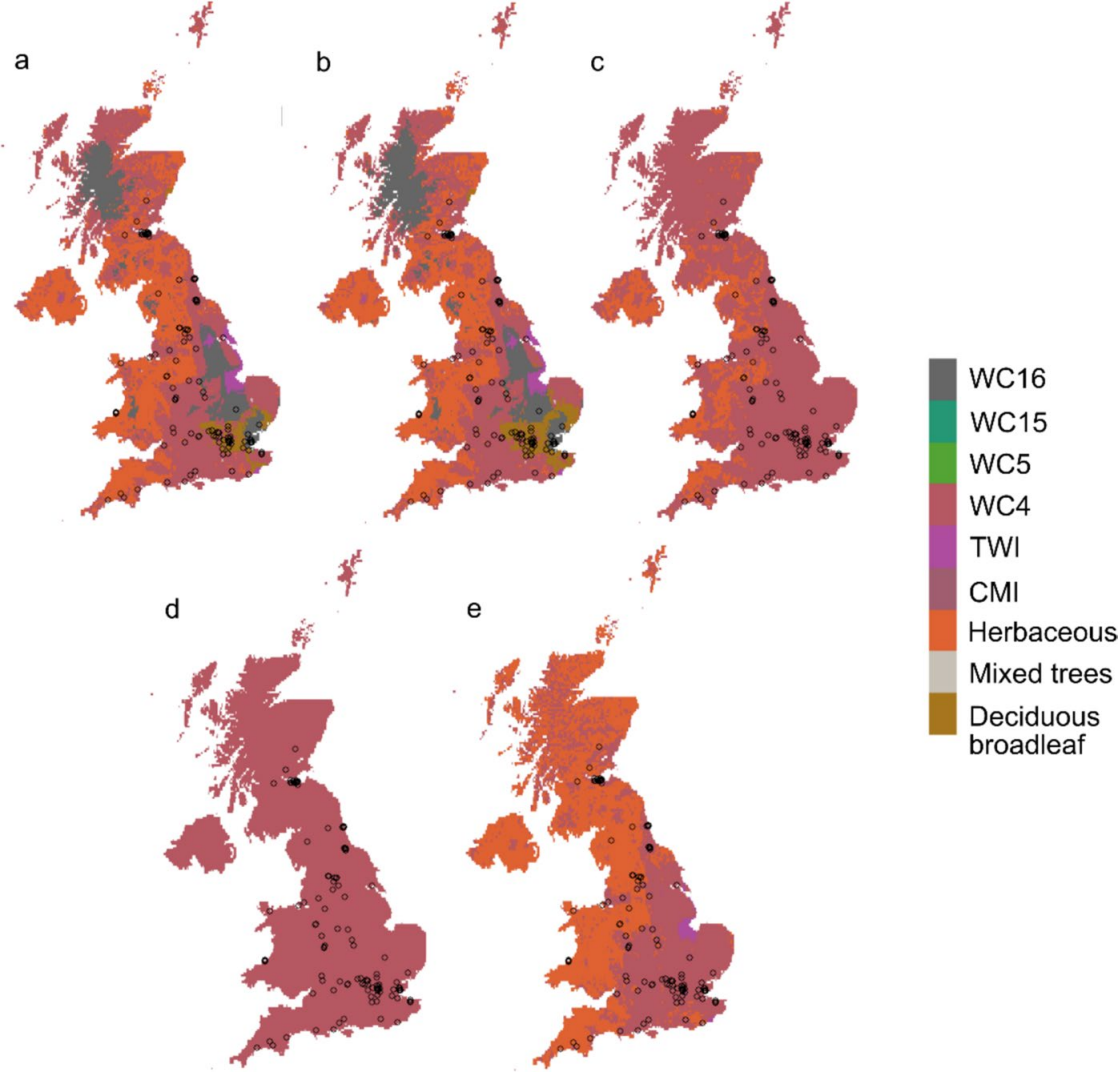
Most different variable to show what is driving main environmental differences between native and invaded range **a** Species-level, **b** Central, **c** Balkan **d** Greek and **e** Italian lineage. Open circles show known UK alpine newt populations

Environmental suitability of the UK (within areas of high confidence and low extrapolation) show large areas of central and eastern England, the Scottish highlands and a small area of Northern Ireland and central Wales to be environmentally suitable for the alpine newt when based on data from both the species-level and central lineage data (Fig. 3a, b). Southwestern England and most of Northern Ireland were predicted to have low suitability, but these fall into areas of lower model confidence due to some extrapolation beyond the limits in which the data was trained (Fig. 3f–g). This extrapolation is largely due to differences in herbaceous cover (Fig. 4 a, b). Evaluation metrics show good model performance for AUC (AUCs above 0.7, Pearce & Ferrier 2000) across top optimised models for both the species-level and central lineage (0.84 and 0.83) but only fair for other metrics (AUC^testBC^ 0.69 species-level, 0.66 central-lineage). Based on TSS values, predictions are considered useful based on the top optimised model (TSS 0.53 and 0.52 for species-level & Central lineage respectively) and are considerably better than random (Allouche et al. 2006) but suboptimal (< 0.4; Zhang et al. 2015) for the species-level and Central lineage test datasets (TSS^testBC^ 0.31). There is some model overfitting, as demonstrated by slightly higher AUC for training data compared to testing (0.15–0.17 AUC^DIFF^ Table 1).

**Table 1:** Evaluation metrics (AUC^tm^, AUC^trainBC^ AUC^testBC^, AUC^diff^, TSS^tm^, TSS^trainBC^, TSS^testBC^), feature classes (q = quadratic, p = power, l = linear, h = hinge) and regularisation for the top-ranking maxnet models per lineage and for species-level analysis

LINEAGE	AUC^tm^	AUC^trainBC^	AUC^testBC^	TSS^tm^	TSS^trainBC^	TSS^testBC^	AUC^diff^	FEATURE CLASSES^tm^	REGULARISATION^tm^
Species-level	0.84	0.84	0.69	0.53	0.53	0.31	0.15	qp	0.1
Central	0.83	0.83	0.66	0.52	0.52	0.31	0.17	qp	0.1
Balkan	0.90	0.90	0.75	0.65	0.67	0.47	0.15	lh	0.7
Greek	0.96	0.95	0.93	0.80	0.78	0.84	0.02	qh	1.3
Italian	0.92	0.94	0.88	0.72	0.80	0.80	0.06	h	2.7

^tm^ refers to the hyperparametrised and optimised top model based on AUC, ^trainBC^ and ^testBC^ refers to block-cross validated training and testing model outcomes, respectively and AUC ^diff^ refers to the difference between training and testing for block-cross validated models

Environmental suitability based on data from the Balkan-lineage shows high suitability up the Pennine mountain range as well as in parts of north east Scotland, northeast and southwest Wales and northern Ireland. The MESS map (Fig. 3h) however, suggests these predictions should be interpreted with caution—there are large differences between the native-range Balkan conditions and that of the UK, driven largely by differences in temperature seasonality (WC04) and herbaceous plant cover (Fig. 4c). Evaluation metrics for the Balkan lineage are generally high across the board (Table 1) but with some model overfitting (0.15 for AUC^DIFF^). The Greek-lineage evaluation metrics are also good (good model discrimination and very marginal overfitting—Table 1) and invasive suitability of the Greek-lineage alpine newt appears to low for most of the UK but high for northwest Scotland and for the very south of England (Fig. 3d). The MESS map again (Fig. 3i), however, shows that this prediction should again be interpreted with caution due to stark differences in environmental conditions between native (Greek) and introduced (UK) ranges, driven predominantly by differences in temperature seasonality (Fig. 4d). Environmental suitability based on predictions from the Italian lineage show low suitability across the UK, with good model metrics (e.g. AUC^tm^ 0.92) but again with high levels of extrapolation placing less confidence in these predictions (Fig. 3j).

**Fig. 5:**
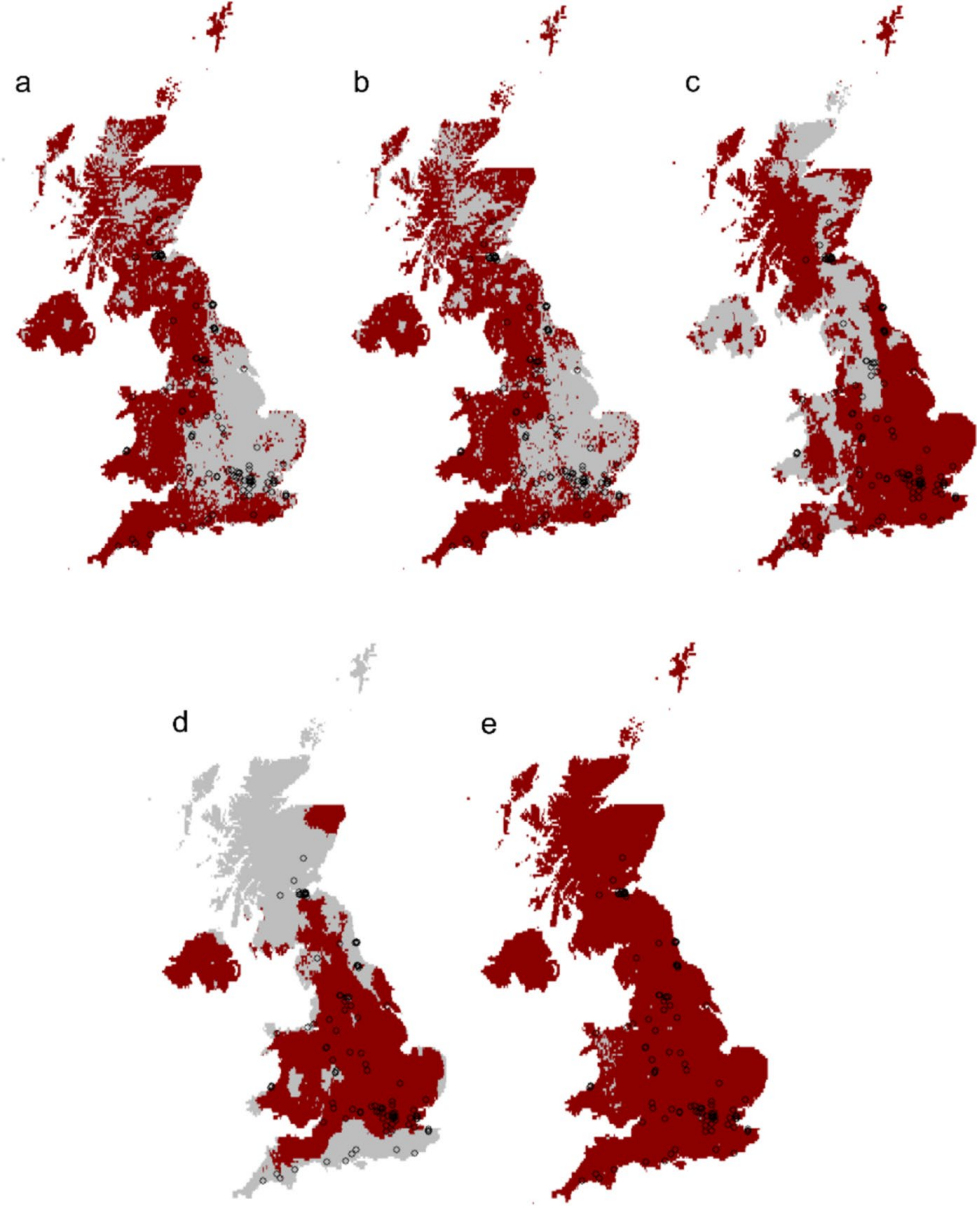
Predicted presence/absence map based on the top ranking optimised and fine-tuned model with the maximum training sensitivity plus specificity threshold for **a** Species-level, **b** Central **c** Balkan **d** Greek and **e** Italian lineage analysis. *Grey* areas are predicted suitable, *red* areas not suitable, and *open circles* are known alpine newt records

Of known UK invasive alpine newt records (rarefied to 1 km to ensure independence of populations n = 113), 66% fell within the area predicted to be environmentally suitable at the species-level, 64% for the central-lineage, 38% for Greek, 12% for Balkans and 0% for Italian (Fig. 5).

### Niche overlap between I. alpestris lineages and known UK records

Pairwise NOT analyses suggests environmental niche equivalence of the Central lineage with the Balkan (*p* = 0.99) and Italian lineages (*p* = 0.78), as well as with the UK records (p = 1). We also found niche equivalence of the Balkan lineage with the Italian lineage (*p* = 0.87). All the equivalence tests for these comparisons had *p*-values > 0.05 and all (except the UK records comparisons with the Central lineage) had at least one significant background test, suggesting some (but limited) statistical power to detect differences if they were present (Table 2). The Central vs Greek lineage is also suggestive of niche equivalence (*p* = 0.41) but a very low niche similarity D value (0.04) and only one significant background test is suggestive of limited power to detect possible differences. Similarly, the Balkan vs UK comparison is suggestive of equivalence (*p* = 0.19) but with a low niche similarity D value (0.08) and no power to detect differences (neither background test is significant). The Greek lineage was significantly different from the Balkan and Italian lineages, as well as the UK records (with all *p*-values for equivalence tests being < 0.04).

**Table 2:** Results of Humboldt niche equivalence and background tests

	Niche similarity (d)	Equivalence p-value	Background 1—> 2 p-value	Background 2—> 1 p-value	Test conclusion
*Niche Overlap test (NOT) of non-analogous environments*					
Central vs Balkan lineages	0.28	0.99	0.004	0.64	No evidence of niche divergence
Central vs Greek lineages	0.04	0.41	0.81	0.02	No evidence of niche divergence. **However, background tests lack power to detect differences**
Central vs Italian lineages	0.09	0.78	0.82	0.004	No evidence of niche divergence
Central lineage vs UK records	0.11	1.0	0.65	0.87	No evidence of niche divergence
Balkan vs Greek* lineages	0.04	0.004	0.57	0.19	**Strong evidence that lineages occupy different niches**
Balkan vs Italian lineages	0.19	0.87	0.44	0.004	No evidence of niche divergence
Balkan lineage vs UK records	0.08	0.19	0.82	0.62	No evidence of niche divergence. **However, background tests have no power to detect differences**
Greek vs Italian lineages*	0.22	0.02	0.004	0.04	**Strong evidence that lineages currently occupy different niches**
Greek lineage vs UK records*	0	0.004	0.004	0.004	**Strong evidence that they currently occupy different niches**
Italian lineage vs UK records*	0	0.004	0.004	0.004	**Strong evidence that they currently occupy different niches**
*Niche Divergence test (NDT) of analogous environments*					
Central vs Balkan lineages	0.28	0.90	0.01	0.79	No evidence of niche divergence
Central vs Greek lineages	0.07	0.09	0.88	0.03	**Weak evidence of niche divergence.** Non-significant equivalence statistic, however on margin of significance
Central vs Italian lineages	0.09	0.71	0.99	0.004	No evidence of niche divergence
Central lineage vs UK	0.17	1.0	0.004	0.87	No evidence of niche divergence
Balkan vs Greek lineages	0.05	0.004	0.72	0.08	**Strong evidence of niche divergence**
Balkan vs Italian lineages	0.22	0.71	0.51	0.004	No evidence of niche divergence
Balkan lineage vs UK records	NA	NA—strong evidence of niche divergence	NA	NA	Not enough localities remain for analysis after removing non-analogous environments. **This, alongside a low niche similarity value (0.08) and no statistical power to detect differences in the NOT is suggestive of niche divergence**
Greek vs Italian lineages	0.58	0.96	0.004	0.005	No evidence of niche divergence
Greek lineage vs UK records	NA	NA—strong evidence of niche divergence	NA	NA	**No analogous environments exist between the Greek lineage and the UK. The absence of shared accessible analogous climates is strong evidence of niche divergence. This is supported by low niche similarity values (0) and significant difference observed in the Niche Overlap Test (0.004)**
Italian lineage vs UK records	NA	NA—strong evidence of niche divergence	NA	NA	**No analogous environments exist between the UK and the Italian lineage. The absence of shared accessible analogous climates is strong evidence of niche divergence. This is supported by low niche similarity values (0) and significant difference observed** **in the Niche Overlap Test (0.004)**

Table includes Niche similarity index (D), and outputs of statistical tests for Niche Overlap test (NOT) of non-analogous environments and Niche Divergence test (NDT) of analogous environments

Subsequent NDT analysis largely match our NOT results, with niche equivalence of the Central lineage with the Balkan and Italian lineages (Table 2). We also find niche equivalence of the Balkan lineage with the Italian lineage. The equivalence tests for these comparisons had *p*-values > 0.05 and significance for at least one of the background tests, suggesting some statistical power to detect differences if they existed (Table 2). Two out of four of the Greek comparisons had strong evidence for niche divergence (Balkan: *p* = 0.004, UK: no comparison was possible between two groups because no shared analogous space existed, as such, Brown and Carnaval (2019) advocated that this be interpreted as strong evidence of niche divergence). The third Greek comparison (between the Greek and Central lineages) resulted in weak evidence of niche divergence with a near significant equivalence *p* value (*p* = 0.09), but with only one significant background test and therefore more limited statistical power to detect differences. The niche of the UK records was found to be strongly divergent from the Balkan, Greek and Italian lineages, but equivalent to the Central lineage.

**Fig. 6:**
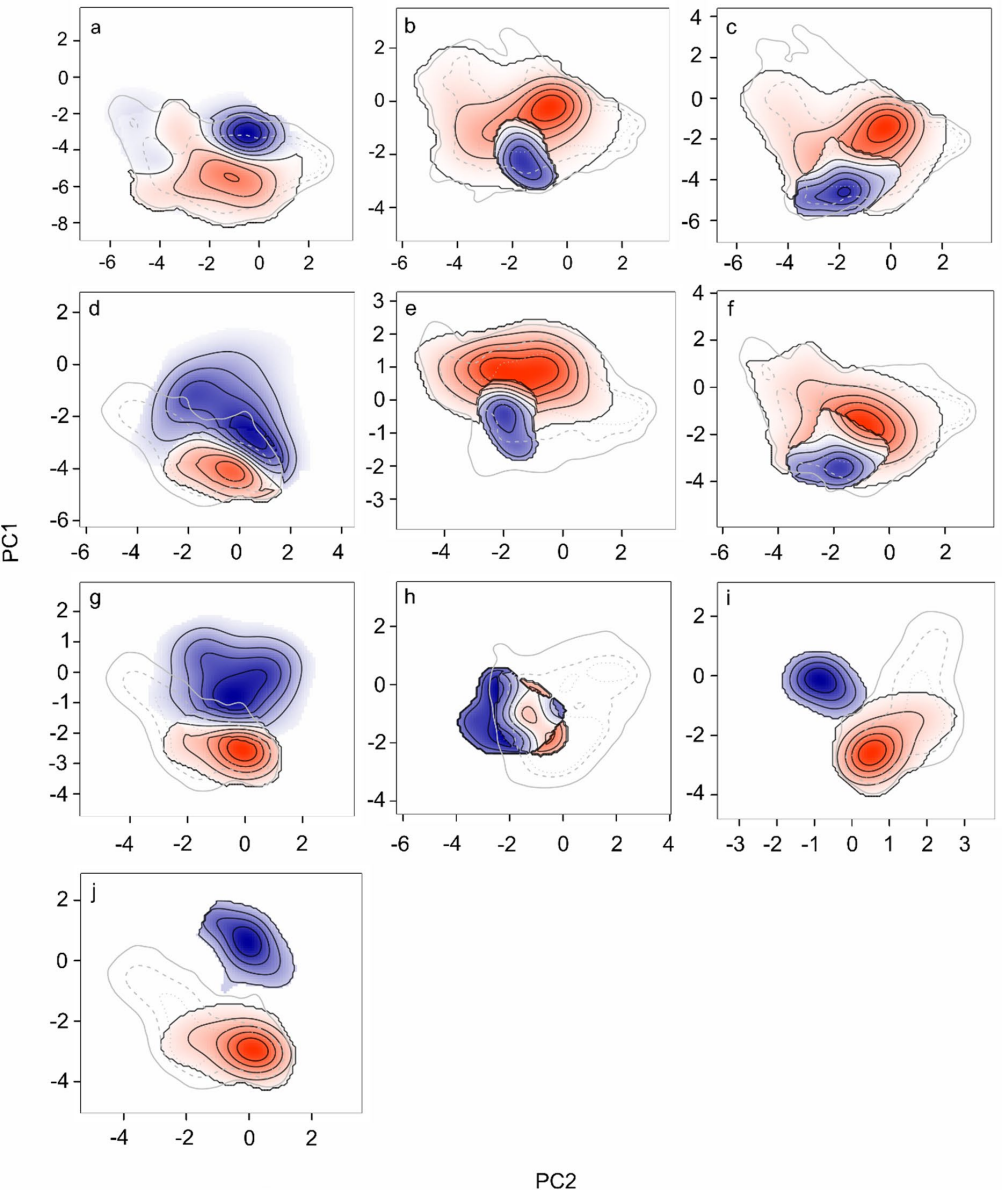
Differences in non-analogous environmental space between **a** Central [*red*] vs Balkan [*blue*] lineage **b** Central [*blue*] vs Greek [*red*] lineage **c** Central [*blue*] vs Italian [*red*] lineage **d** Central [*red*] lineage vs UK records [*blue*] **e** Balkan [*blue*] vs Greek [*red*] lineage **f** Balkan [*blue*] vs Italian [*red*] lineage **g** Balkan [*red*] lineage vs UK [*blue*] records **h** Greek [*blue*] vs Italian [*red*] lineage **i** Greek [*red*] lineage vs UK [*blue*] records **j** Italian [*red*] lineage vs UK [*blue*] records. Darker colours depict areas where the specified environmental space is more abundant, and lighter colours less abundant. *Grey lines* show the environmental space of environment 1 (linked to the *red* lineage). The *black lines* are isopleths that represent differences between the environmental space of the two lineages being compared

**Fig. 7:**
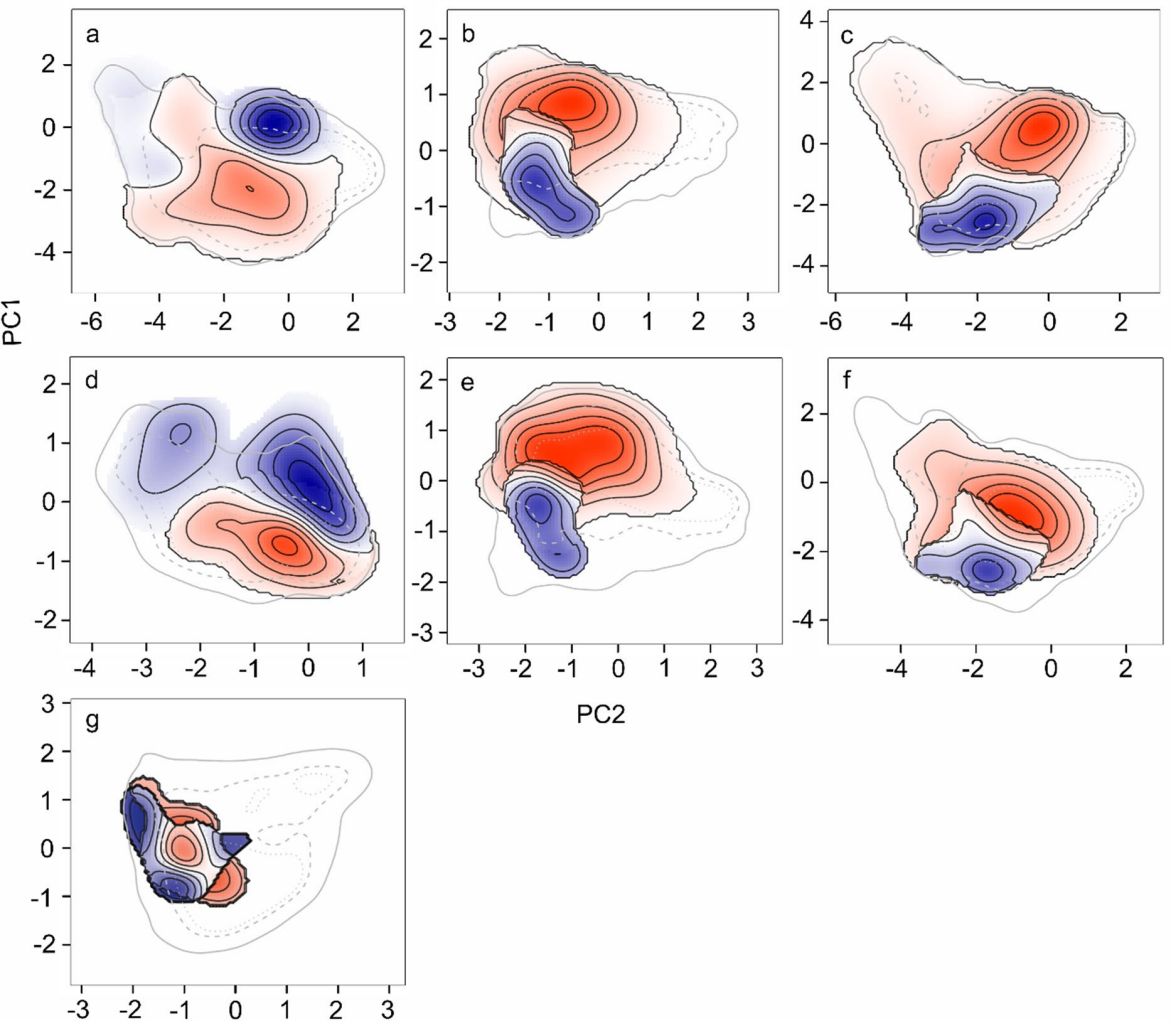
Differences in analogous environmental space between **a** Central [*red*] vs Balkan [*blue*] lineage **b** Central [*blue*] vs Greek [*red*] lineage **c** Central [*blue*] vs Italian [*red*] lineage **d** Central [*red*] lineage vs UK [*blue*] records **e** Balkan [*blue*] vs Greek [*red*] lineage **f** Balkan [*blue*] vs Italian [*red*] lineage **g** Greek [*blue*] vs Italian [*red*] lineage. Darker colours depict areas where the specified environmental space is more abundant, and lighter colours less abundant. *Grey lines* show the environmental space of environment 1 (linked to the *red* lineage). The *black lines* are isopleths that represent differences between the environmental space of the two lineages being compared

Similarities and differences between lineages in non-analogous occupied environmental space can be visualised in Fig. 6a–j, and in analogous space in Fig. 7a–g. Figure 6a–j illustrates how certain lineages occupy distinct environmental conditions outside their shared environmental range, with some having larger visual differences than others. The Greek lineage vs UK records and Italian lineage vs UK records, for example, both had the smallest niche similarity values (of zero), and have least visual overlap on plots Fig 6i–j. These, alongside the Balkan lineage vs UK records could not be plotted for the NDT comparisons due to lack of analogous environmental space. Figure 7a–g focuses on analogous environmental space, where comparisons are restricted to environmental conditions shared by both lineages being evaluated. Together, the results indicate that while some lineages, such as the Greek lineage, demonstrate significant divergence across multiple lineages in both analogous and non-analogous space, others, like the Central lineage, show considerable overlap. The percentage explained by the principal components was high but varied across comparisons; Central vs Balkan lineage: PC1 = 40.01% PC2 = 17.09%, Central vs Greek lineage: PC1 = 29.11% PC2 = 22.61%, Central vs Italian lineage: PC1 = 38.39%, PC2 = 19.48%, Central lineage vs UK records: PC1 = 43.34%, PC2 = 22.95%, Balkan vs Greek lineage: PC1 = 37.06% PC2 = 21.86%, Balkan vs Italian lineage: PC1 = 43.51% PC2 = 16.81%, Balkan lineage vs UK records: PC1 = 43.94%, PC2 = 23.48%, Greek vs Italian lineage: PC1 = 36.5% PC2 = 22%, Greek lineage vs UK records PC1 = 41.52% PC2 = 24.57%, Italian vs UK lineage: PC1 = 38.34% PC2 = 25.77%.

## Discussion

Our aims were to determine the environmental suitability of the UK for a non-native species of growing prevalence, to test if invasive suitability differed by evolutionary lineage and to ascertain whether any differences in invasive suitability were due to environmental niche divergence. We used species distribution and ecological niche models in a two-step process and our findings suggest good environmental suitability in parts of the UK for the alpine newt. Evidence of niche divergence for some evolutionary lineages (most notably the Greek), suggest the differences in lineage invasibility (as shown by SDM predictions) could be of note, but vastly different environments and therefore high levels of extrapolation for Greek, Balkan and Italian lineages suggest species-level predictions are currently the most robust unit of analysis, despite only 66% of non-native UK records falling within areas predicted to be suitable. Our findings provide a baseline for further risk analyses and a series of recommendations for further research into the environmental suitability of the UK for the alpine newt.

At the species-level, our findings predict several regions of the UK to be of high environmental suitability for the alpine newt, with the most suitable regions and areas of high model certainty (i.e. low MESS extrapolation) being central and eastern England and central and northern Scotland, with good support across most evaluation metrics. High suitability for this species in these areas could have conservation implications for both native amphibian communities and for the alpine newt itself. Empirical evidence of ecological impacts of the alpine newt on native species is currently in its infancy, but disease transmission, competition and predation have all been cited as potential threats (Bell 2016). Patterns of *Bd* occurrence in the UK are strongly linked with the presence of non-native amphibians (Cunningham and Minting 2008) and endemism of *Bd* has been suggested for alpine newts in northern Europe (Spitzen-Van Der Sluijs et al. 2014). Similarly, subclinical *Ranavirus* infections have been detected in alpine newts at an introduced population in Catalonia (Martínez-Silvestre et al. 2017), and broad-scale associations have been found between the occurrence of newts and increased prevalence of ranavirosis in common frogs in the UK (North et al. 2015). Alpine newts are effective predators of common frog spawn (Denoël and Demars 2008; Sztatecsny et al. 2013), will predate heterospecific and intraspecific egg and larvae (Mettouris and Giokas 2017) and can be competitively successful alongside smaller bodied newts (Hloušková et al. 2018; Winterová and Gvoždík 2018).

Future work could fine-tune risk predictions by focussing on invasive-range data only and anthropogenic variables, which have been shown to be important determinants of freshwater invasive species range distribution (Rodríguez-Rey et al. 2019). We were unable to explore variables associated with human activity here due to our focus on native-range data and the opposing relationship of human-disturbance factors in native versus invaded distributions (i.e. often constricting dispersal in native ranges and enabling spread in invasive ranges) (Pimm et al. 2014; Rodríguez-Rey et al. 2019). Investigating how these risks change with climate breakdown will be important (Porfirio et al. 2014) since an understanding of where invasion risks may be more geographically focused can be combined with expert elicitation to streamline conservation efforts in ongoing iterative decision-making processes (Sofaer et al. 2019).

With 66% of known UK records falling within environmentally suitable areas at the species-level, a series of testable hypotheses could be generated to further understand the invasion ecology of this species. We currently know that the UK populations contain both the Central and Italian lineages, but so far only a small proportion of UK populations have been genotyped (Ball et al. 2023; Robbemont et al. 2023). Genotyping of each established population in the UK could help ascertain the level of niche conservatism between native lineages and their invaded ranges since equivalence tests could be used if the lineage of each invaded alpine newt population was known. Strubbe et al. (2015a) found that niche shifts were rare and niche unfilling common between native and invaded ranges (including amphibians) and further investigation could elucidate this for alpine newts in the UK. Genotyping could also help ascertain whether the records falling outside of high suitability areas are of differing lineage or have differing ability to spread from introduction points. For example, mismatches between predicted niche and geographic distribution in Proteaceae plants were linked with dispersal ability, persistence and time-lags in local extinction (Pagel et al. 2020). Alpine newt evolutionary lineages may have differing abilities to sustain and disperse, resulting in some isolated populations that could either be highly persistent or unviable sink populations (Pulliam 1988) in the process of becoming locally extinct (Pagel et al. 2020). Populations outside of areas predicted to be suitable may also be found in pockets of highly suitable habitat, or conditions that could not be detected at the spatial resolution of the data. For example, errors of omission for Ixodid tick species in Florida were found within a specific habitat that was too narrow to be detected at the 1km scale in which analysis was conducted (Glass et al. 2021). Habitat and genetic studies of alpine newt records falling outside of environmentally suitable areas could help test these hypotheses.

The evolutionary history of the alpine newt is complex (Pabijan and Babik 2006; Recuero et al. 2014; Chiocchio et al. 2017; Vörös et al. 2021), sub-lineages are still being discovered (Vörös et al. 2021), life histories and morphologies are geographically varied (Denoël et al. 2001; Ivanović et al. 2009; Vukov et al. 2011) and its range covers three prominent glacial refugia (Schmitt 2007). This, together with the fact that environmental variables such as climate are important for amphibian function and physiology (Feder and Burggren 1992), behaviour (Denoël et al. 2005b), abundance (Canestrelli et al. 2006) and distribution (Araújo and Peterson 2012), make differences in environmental niche across lineages unsurprising. Here we found evidence of niche divergence of the Greek lineage from both the Central and Balkan lineages, as well as divergence of the Greek, Balkan and Italian lineages from the niche of the UK non-native records of the alpine newt. Notably, the Central lineage seems to possess equivalence in Grinellian niche with the UK which could indicate a predominant Central lineage provenance of invaded populations.

Time estimates for the evolution of alpine newt subspecies based on maximum clade credibility trees suggest *ichthyosaura alpestris veluchiensis* to be the oldest subspecies (Recuero et al. 2014). This may help explain the pattern of divergence found in our analyses, since this subspecies falls to the Greek lineage (Recuero et al. 2014) and we might expect in some instances for taxa with a more distant shared history to have more dissimilar niche and vice versa (Burns and Strauss 2012; Tromas et al. 2018). Greece is a rugged mountainous archipelago with a distinct Mediterranean climate. This, in combination with its role as glacial refugia in the last ice age (Leontaritis et al. 2020) could have contributed to niche divergence in isolated populations over time. Differing niches of the Balkan, Italian and Greek lineage compared to the UK is perhaps unsurprising given the level of extrapolation of environmental variables seen in our maxnet analysis. Several of our niche overlap and divergence analyses suffered from insignificant background tests and this reduced power could have influenced our ability to detect differences in other comparisons. Some of this is likely due to the huge geographic areas in which some of the lineages can be found (e.g. across central Europe) and hence broad array of environmental conditions within them, coupled with the fact that some lineages possessed limited spatially unique occurrence records. A mixture of differing data sharing policies, citizen science programmes and funding availability could reflect the geographic biases seen in open-access data depositories such as GBIF (Feldman et al. 2021) and this should be addressed as a matter of urgency.

Together, our species distribution model predictions and niche divergence tests suggest lineage probably does matter for invasive suitability in the UK and this could have implications for invasion risk assessments, trade biosecurity and quarantine measures, as suggested for ring-necked parakeets and bark beetles (Cardador et al. 2016; Godefroid et al. 2016). However, due to vastly differing environments between the UK and the Greek, Balkan and Italian lineages (and hence extrapolation beyond the values distribution models were trained on), a precautionary approach may be considered for spatial risk assessments of this species. Species-level predictions remain the most robust level of analysis based on the best data currently available but additional analysis will be important as further data becomes available.

When possible, higher taxonomic resolution predictions will be useful since the Central lineage in particular spans a large geographic area and is made up of two subspecies (Recuero et al. 2014) that could have differing environmental requirements. The configuration of subspecies for alpine newts is complex and discussion is ongoing around groupings at the species-level (Raffaëlli 2018; Speybroeck et al. 2020). Predictions made on unresolved taxonomic classifications can be damaging to conservation efforts as shown by the pygmy newt *Triturus pygmaeus* and marbled newt *Triturus marmoratus* (Romero et al. 2014). These were previously recognised as subspecies of *Triturus marmoratus* and differing predicted responses of each species to future climate scenarios (that in the past had been considered together) showed that the vulnerability of one could be masked by low climate change vulnerability of the other (Romero et al. 2014). New insights into within-subspecies reproductive isolation will help resolve discussions around alpine newt taxonomy (Speybroeck et al. 2020) and aid finer resolution comparisons of niche and invasive abilities in the future.

Our characterisation of evolutionary lineages are based on best current knowledge of broad scale patterns as outlined by Recuero et al. (2014) but finer geographic scale and genotyped data that may be available in the future could provide greater insights and intricacies at lineage boundaries. Research by Vörös et al. (2021) and Chiocchio et al. (2017), for example, improves our understanding of the evolutionary history of alpine newts at finer geographic scales, but also highlights potential difficulties for defining lineages geographically at evolutionary contact zones since phylogeographic structure is often complex at lineage or species boundaries (Vences and Wake 2007; Dufresnes et al. 2019; Pyron et al. 2022). Genetic sequencing across a larger geographic area and multiple genetic markers (mitochondrial and nuclear) could help assign distribution data to lineage at finer geographic scales, and this would be especially useful for both contact zones and broad geographic areas such as the Central lineage.

With significant increases of newly established non-native species forecast by the mid-twenty-first century (Seebens et al. 2021), environmental suitability predictions are increasingly useful tools for identifying where an introduced species may thrive and where, therefore, conservation efforts and resources should be focused. An understanding of the likelihood of establishment and spread are amongst a minimum set of criteria suggested for thorough risk assessments of introduced species. Roy et al. (2018) emphasise the importance of such exercises despite scarce data, so long as uncertainty is acknowledged. The use of species distribution and ecological niche models are common contributors to risk analyses (Srivastava et al. 2019) but only recently has the importance of considering phylogeny been fully realised (Smith et al. 2019). Despite the validity in comparing invasiveness at varying evolutionary or biological units, there are challenges to this approach when data are limited or unevenly available or environments are vastly different between native and invaded ranges, as demonstrated here for the alpine newt. Our findings suggest that species-level analyses are currently the most robust level at which to judge risk for this species, but further work is needed to ascertain why this doesn’t fully capture the distribution of known alpine newt records in the UK. We have generated testable hypotheses and indicate the role of a range of environmental variables for the successful invasion of a small urodele. We provide a baseline for a range of further work but most importantly show that parts of the UK are currently well within the environmental niche of the alpine newt, with potential – but currently unquantified—implications for the conservation of native amphibian communities.

## Supplementary Information

Below is the link to the electronic supplementary material.

Supplementary file1 (CSV 400 kb)

Supplementary file2 (TXT 115 kb)

## Data Availability

The collated alpine newt distribution dataset and code utilised in this study is available in the supplementary information and environmental datasets are available online from links found within the methods section of this manuscript.
